# Neutralization of N501Y mutant SARS-CoV-2 by BNT162b2 vaccine-elicited sera

**DOI:** 10.21203/rs.3.rs-143532/v1

**Published:** 2021-01-13

**Authors:** Pei-Yong Shi, Xuping Xie, Jing Zou, Camila Fontes-Garfias, Hongjie Xia, Kena Swanson, Mark Cutler, David Cooper, Vineet Menachery, Scott Weaver, Philip Dormitzer

**Affiliations:** The University of Texas Medical Branch at Galveston; University of Texas Medical Branch; University of Texas Medical Branch; University of Texas Medical Branch; University of Texas Medical Branch; Pfizer; Pfizer; Pfizer; The University of Texas Medical Branch; The University of Texas Medical Branch at Galveston; Seqirus

**Keywords:** SARS-CoV-2, COVID-19, vaccines

## Abstract

Rapidly spreading variants of SARS-CoV-2 that have arisen in the United Kingdom and South Africa share the spike N501Y substitution, which is of particular concern because it is located in the viral receptor binding site for cell entry and increases binding to the receptor. We generated isogenic N501 and Y501 SARS-CoV-2. Twenty human sera from the mRNA-based vaccine BNT162b2 trial exhibited equivalent neutralizing titers to the N501 and Y501 viruses.

We previously reported that BNT162b2, a nucleoside modified RNA vaccine that encodes the SARS-CoV-2 full length, prefusion stabilized spike glycoprotein (S), elicited dose-dependent SARS-CoV-2–neutralizing geometric mean titers (GMTs) that were similar to or higher than the GMT of a panel of SARS-CoV-2 convalescent human serum samples.^1^ We subsequently reported that, in a randomized, placebo-controlled trial in approximately 44,000 participants 16 years of age or older, a two-dose regimen of BNT162b2 conferred 95% protection against Covid-19.^2^

Since the previously reported studies were conducted, rapidly spreading variants of SARS-CoV-2 have arisen in the United Kingdom and South Africa.^3,4^ These variants have multiple mutations in their S glycoproteins, which are key targets of virus neutralizing antibodies. These rapidly spreading variants share the spike N501Y substitution. This mutation is of particular concern because it is located in the viral receptor binding site for cell entry, increases binding to the receptor (angiotensin converting enzyme 2), and enables the virus to expand its host range to infect mice.^5,6^

We generated an isogenic Y501 SARS-CoV-2 on the genetic background of the N501 clinical strain USA-WA1/2020 (Supplementary Fig. 1), which also provided the genetic background of the BNT162b2-encoded spike antigen. The N501 and Y501 viruses had similar plaque morphologies on Vero E6 cells (Supplementary Fig. 2). Sera of 20 participants in the previously reported trial,^1,2^ drawn 2 or 4 weeks after immunization with two 30-mg doses of BNT162b2 spaced three weeks apart (Supplementary Fig. 3), were tested for neutralization of N501 and Y501 viruses by a 50% plaque reduction neutralization assay (PRNT_50_). The neutralization titers against the Y501 virus are equivalent or slightly higher than those against the N501 virus (Fig. 1 and Supplementary Table 1). The ratio of the 50% neutralization GMT of the sera against the Y501 virus to that against the N501 virus was 1.46 (Supplementary Fig. 4), indicating no reduction in neutralization activity of vaccines against the virus bearing the Y501 spike.

A limitation of this finding is that the Y501 virus does not include the full set of spike mutations found on the rapidly spreading strains in the UK or South Africa.^3,4^ Nevertheless, preserved neutralization of Y501 virus by BNT162b2 vaccine-elicited human sera is consistent with preserved neutralization of a panel of 15 pseudoviruses bearing spikes with other mutations found in circulating SARS-CoV-2 strains.^7^

The ongoing evolution of SARS-CoV-2 necessitates continuous monitoring of the significance of changes for vaccine coverage. This surveillance should be accompanied by preparations for the possibility that a future mutation in SARS-CoV-2 might necessitate a vaccine strain change. Such a vaccine update would be facilitated by the flexibility of mRNA-based vaccine technology.

## Methods

### Construction of isogenic viruses.

We prepared an isogenic pair of SARS-CoV-2 containing the N501 or Y501 spike protein (Figure S1). The N501Y mutation was generated by an A-to-T substitution at nucleotide 23,063 of the viral genome using an infectious cDNA clone of clinical strain WA1 (2019-nCoV/USA_WA1/2020).^1^ Following a previously reported mutagenesis protocol,^2^ we recovered N501 and Y501 viruses with titers of >10^7^ plaque-forming units (PFU) per ml. The two viruses developed similar plaque morphologies on Vero E6 cells (Fig. S2).

### Serum specimens and neutralization assay.

The immunization and serum collection regimen is illustrated schematically in Fig. S3. For measuring neutralization titers, each serum was 2-fold serially diluted in culture medium with the first dilution of 1:40 (dilution range of 1:40 to 1:1280). The diluted serum was incubated with 100 PFU of N501 or Y501 virus at 37 °C for 1 h, after which the serum-virus mixtures were inoculated onto Vero E6 cell monolayer in 6-well plates. A conventional (non-fluorescent) plaque reduction neutralization assay was performed to quantify the serum-mediated virus suppression as previously reported.^3^ A minimal serum dilution that suppressed >50% of viral plaques is defined as PRNT_50_. A table of the neutralization titers is provided (Table S1). The ratio for each serum of the PRNT_50_ against N501 and Y501 virus is plotted in Fig. S4.

## Declarations

### Data availability

The data that support the findings of this study are available from the corresponding authors upon reasonable request.

## Supplementary Material

Supplement
