# Supplementary material for: Neutralization of N501Y mutant SARS-CoV-2 by BNT162b2 vaccine-elicited sera
Source: Res Sq. 2021 Jan 13:rs.3.rs-143532. Preprint. [Version 1] doi: 10.21203/rs.3.rs-143532/v1 (PMC7814835; doi:10.21203/rs.3.rs-143532/v1)
Supplement: Supplement [file 3c1941e1a3c5b5c9576e4eb3.pdf]

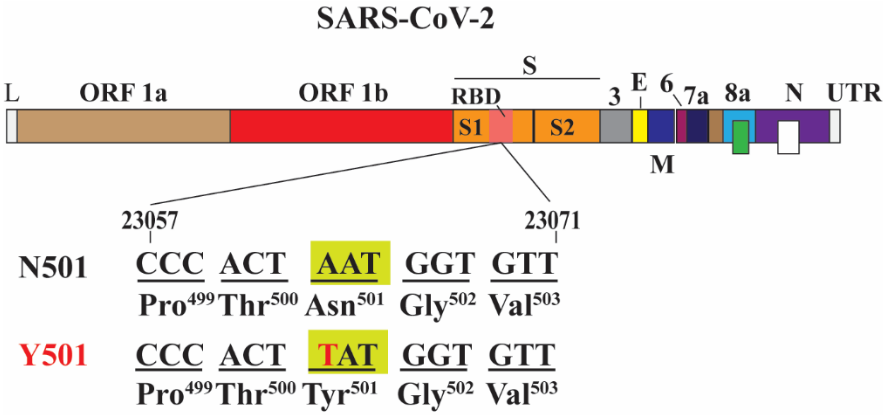

Supplementary Figure 1. Diagram of the N501Y substitution. L – leader sequence; ORF – open reading frame; RBD – receptor binding domain; S – spike glycoprotein; S1 – N-terminal furin cleavage fragment of S; S2 – C-terminal furin cleavage fragment of S; E – envelope protein; M – membrane protein; N – nucleoprotein; UTR – untranslated region.

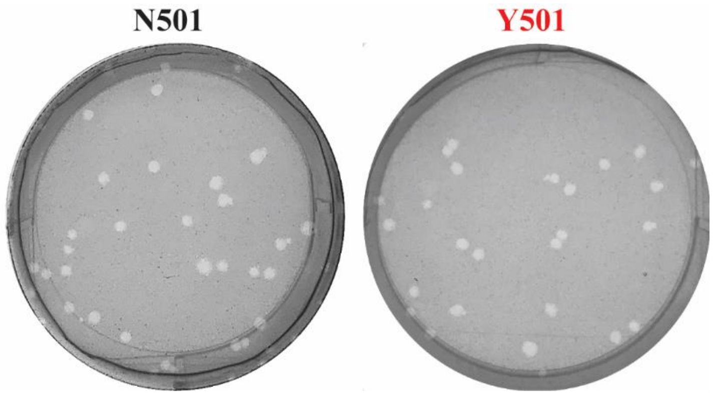

Supplementary Figure 2. Plaque morphologies of N501 and Y501 SARS-CoV-2 on Vero E6 cells.

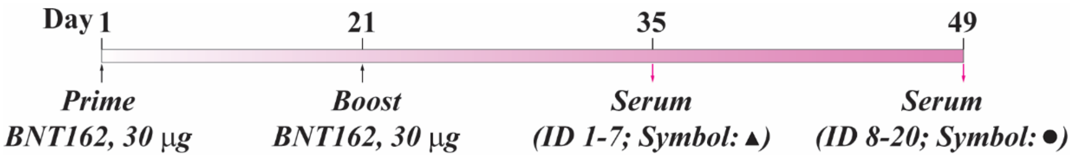

Supplementary Figure 3. Scheme of the BNT162 vaccination and serum sampling.

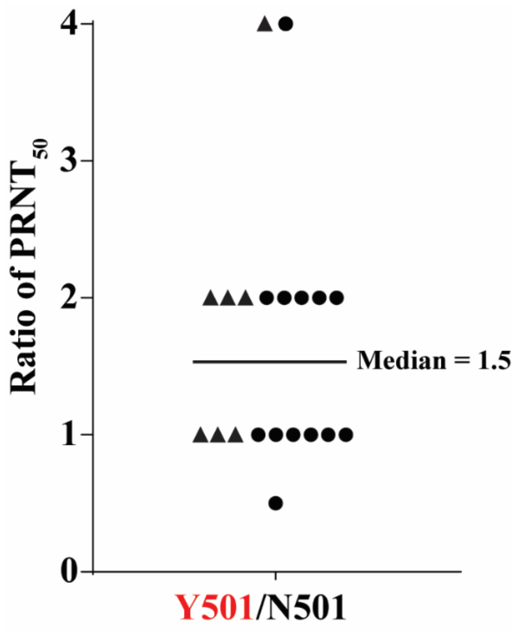

Supplementary Figure 4. Plot of the ratio of $PRNT_{50}$ between Y501 and N501 viruses. Triangles represent sera drawn two weeks after the second dose; circles represent sera drawn four weeks after the second dose.

Supplementary Table 1. $PRNT_{50}$ values of 20 BNT162b2 post-immunization sera against N501 and Y501 SARS-CoV-2.

Serum ID	$PRNT_{50}$		$PRNT_{50}$ ratio (Y501/N501)
	N501	Y501	
1	160	640	4
2	160	320	2
3	320	640	2
4	80	160	2
5	160	160	1
6	320	320	1
7	640	640	1
8	160	160	1
9	640	640	1
10	640	1280	2
11	160	640	4
12	320	320	1
13	640	1280	2
14	640	320	0.5
15	320	640	2
16	320	640	2
17	640	640	1
18	640	1280	2
19	640	640	1
20	640	640	1
